# Supplementary material for: Sparse recovery of undersampled intensity patterns for coherent diffraction imaging at high X-ray energies
Source: Sci Rep. 2018 Mar 21;8:4959. doi: 10.1038/s41598-018-23040-y (PMC5862902; doi:10.1038/s41598-018-23040-y)
Supplement: Supplementary file 1 — Supplementary Material [file 41598_2018_23040_MOESM1_ESM.pdf]

Sparse recovery of undersampled intensity patterns for coherent diffraction imaging at high X-ray energies (SUPPLEMENTARY MATERIAL)

S. Maddali[a,*], I. Calvo-Almazan[a], J. Almer[b], P. Kenesei[b], J. S. Park[b], R. Harder[b], Y. Nashed[c,d], S. O. Hruszkewycz[a]

[a]*Materials Science Division, Argonne National Laboratory, Lemont IL 60439 (USA)*
[b]*X-ray Science Division, Argonne National Laboratory, Lemont IL 60439 (USA)*
[c]*Mathematics & Computer Science Division, Argonne National Laboratory, Lemont IL 60439 (USA)*
[d]*Department of Electrical Engineering & Computer Science, Northwestern University, Evanston IL 60208 (USA)*

Abstract

This document is the Supplementary Material for "Sparse recovery of undersampled intensity patterns for coherent diffraction imaging at high X-ray energies ". This document contains:

1. A rigorous proof of the fact that a system of linear constraints obtained by translating a detector with a finite pixel size in its plane is necessarily underdetermined.
2. A rigorous proof that each image in a Bragg coherent diffractive imaging (BCDI) data set is necessarily band-limited.
3. An evaluation of the sparse recovery technique described in the main text in the presence of Poisson noise.
4. An evaluation of the sparse recovery with different upsampling strategies.

1. Detector-plane upsampling: an underdetermined system of equations

Our goal is a method to reverse the binning process described in Section 2 of the main text and re-obtain the original high-resolution representation of a single detector image.

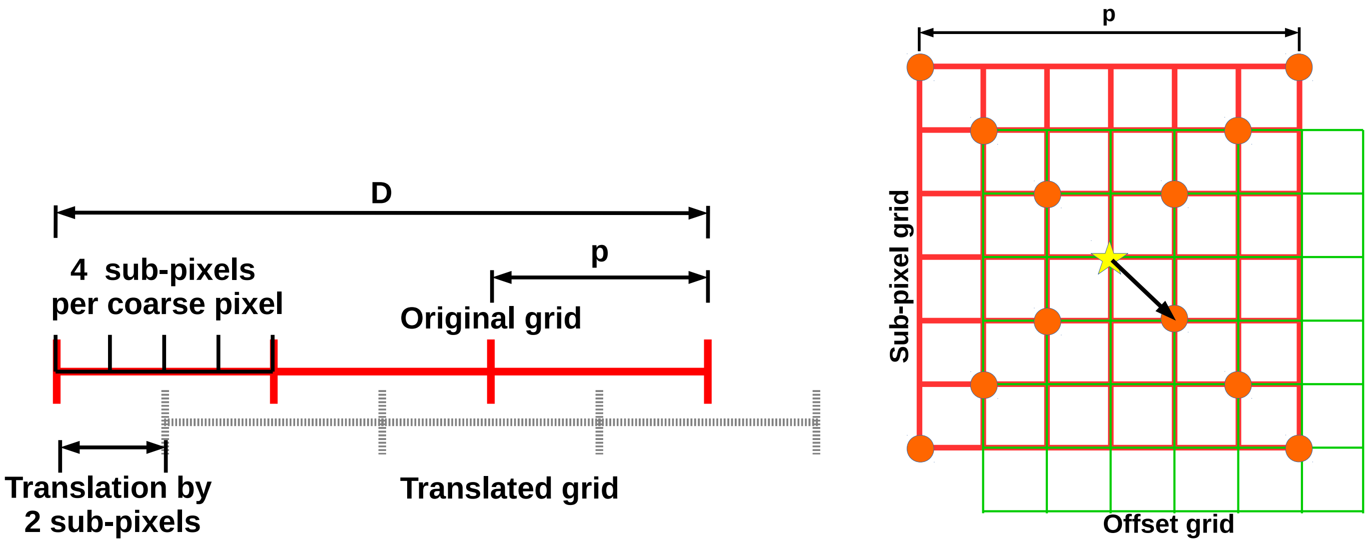

(a) (b)

Figure 1: **(a)** Example of one-dimensional grid offsets in steps of a quarter of a physical pixel. **(b)** Two-dimensional grid offsets in our simulations along the physical pixel diagonals. The star signifies the centre of the un-translated detector, while the orange markers denote the centres of the offset detector.

In a high-energy BCDI experiment, the fine pixel size is determined by the smallest offset of the detector as a matter of convenience. Offsets in fractions of the chosen fine pixel size are undesirable since one is forced to make assumptions about the intensity distribution within a single fine pixel. Under such sampling conditions, we can obtain constraints (shown in the main text Figure 2) that couple the intensities of the fine "sub-pixels" with the measured coarse pixel intensities. We show in this section that these sub-pixels always outnumber the constraints available through detector translation, regardless of the fineness of the offset. In other words, the system of equations that couples the sub-pixels to coarse pixel measurements

*Corresponding author
Email address: `smaddali@anl.gov` (S. Maddali)

is always underdetermined, and has no unique solution. Some form of additional knowledge of the system is required to adequately constrain it.

To demonstrate the underdetermined nature of the problem, we consider one-dimensional binning for simplicity (refer to Figure 1a). Suppose we wish to upsample to a resolution of m sub-pixels per coarse pixel. Here, m is simply the pixel binning factor (PBF) of our high-energy simulations, and in our figure, $m = 4$. If the original aperture size is D with a pixel size p, the number of pixels is $N \equiv D/p$. The periodicity of the binning grid implies that we need only offset the detector to $m-1$ sub-pixel positions to the right in order to generate unique constraints. Any further offset would simply result in redundant constraints. Any offset to the left can also be expressed as an equivalent offset to the right because of this periodicity. Since we are only interested in the range of reciprocal space defined by the original aperture D (red grid), we discard coarse pixel measurements that lie partially outside this region. The total number of unique constraints is therefore: $M = N + (m-1)(N-1)$, where N constraints come from the un-translated grid and each of the $m-1$ grid offsets contribute $N-1$ constraints. On the other hand, the number of sub-pixels coupled by these constraints is $U = m \times N$.

These numbers generalise to higher dimensions in a straightforward manner: $M_d = [N + (m-1)(N-1)]^d$ and $U_d = (mN)^d$ where d is the dimensionality of the space. The ratio of constraints to unknown quantities is therefore:

$$\sigma_d = \frac{M_d}{U_d} = \left[1 - \frac{1}{N} + \frac{1}{mN}\right]^d < 1 \tag{1}$$

For two-dimensional images, $d = 2$. Further, in the limit of infinitesimal detector offsets, $m \to \infty$ and $\sigma_d \to (1 - 1/N)^d = (1 - p/D)^d$, which is always < 1.

Thus, a finite coarse pixel size p ensures that M_d is always less than U_d and therefore the system of equations for the sub-pixels is always underdetermined and without a unique solution unless additional knowledge about the system is incorporated. As an example, non-negativity could be enforced on the sub-pixel intensities. In our methodology, the additional information is provided through the fact that the diffracted intensity pattern of a compact crystal has compact support in real space. This physical insight applies to all compact single crystals and when successfully incorporated into the system of equations, paves the way for the use of sparse numerical solvers in high-energy BCDI.

In our simulations, the detector was offset along both diagonals of the coarse pixels (Figure 1b). Diagonal offsets ensure coupling between all sub-pixels in the two-dimensional grid, as opposed to purely horizontal or vertical offsets. For example, purely horizontal offsets restrict the sub-pixel coupling to the horizontal 'bands' traced by each row of coarse pixels, with no coupling to the bands above or below. An analogous argument holds for vertical offsets. Only a combination of horizontal and vertical (*i.e.* diagonal) offsets ensures coupling between all the sub-pixels.

Counting all possible constraints in the case of diagonal offsets is straightforward: if m is odd, then there are $2m-1$ unique detector positions along the diagonals and if m is even, there are $2m$ positions along the diagonals. In both cases, exactly one detector position (the zero-offset) contributes N^2 constraints, while the remaining contribute $(N-1)^2$ constraints. Thus the maximum number of unique constraints for diagonal offsets is given by:

$$M = \begin{cases} N^2 + (2m-1)(N-1)^2 & \text{if } m \text{ is even} \\ N^2 + (2m-2)(N-1)^2 & \text{if } m \text{ is odd} \end{cases} \tag{2}$$

2. Proof of signal sparsity

The projection-slice theorem [1, 2] states that any two-dimensional slice (S) of the three-dimensional Fourier transform (F_3) of a scalar field $f(\mathbf{x})$ can be alternately obtained by taking the two-dimensional Fourier transform (F_2) of the projection (P_S) of $f(\mathbf{x})$ in the slicing plane:

$$S \cdot F_3 f(\mathbf{x}) = F_2 \cdot P_S f(\mathbf{x}) \tag{3}$$

If $f(\mathbf{x})$ is specifically the Patterson function of the scattering from a compact single crystal, then the LHS of Equation (3) is the quantity that is measured on an area detector at each point in the rocking curve. Computing the 2D inverse Fourier transform throughout, we have:

$$F_2^{-1}\left[S \cdot F_3 f(\mathbf{x})\right] = F_2^{-1} F_2 P_S f(\mathbf{x}) = P_S f(\mathbf{x}) \tag{4}$$

The LHS of Equation (4) is the Fourier representation of a single detector image indexed by the slicing plane S, while the RHS is a planar projection of a compact Patterson function, and therefore is also compact. The sparsity of the two-dimensional Fourier transform is ensured through provision of a sufficient buffer region as described in Section 3 of the main text. Thus *each detector image* in a Bragg CDI experiment has a sparse representation, making compressed sensing techniques applicable to each one independently.

3. Detector noise

In this section we address the issue of sparse recovery of high-energy diffraction images in the presence of simulated detector noise. We repeat the SRTF computation from Section 4 of the main text for two layers of the simulated ground truth,

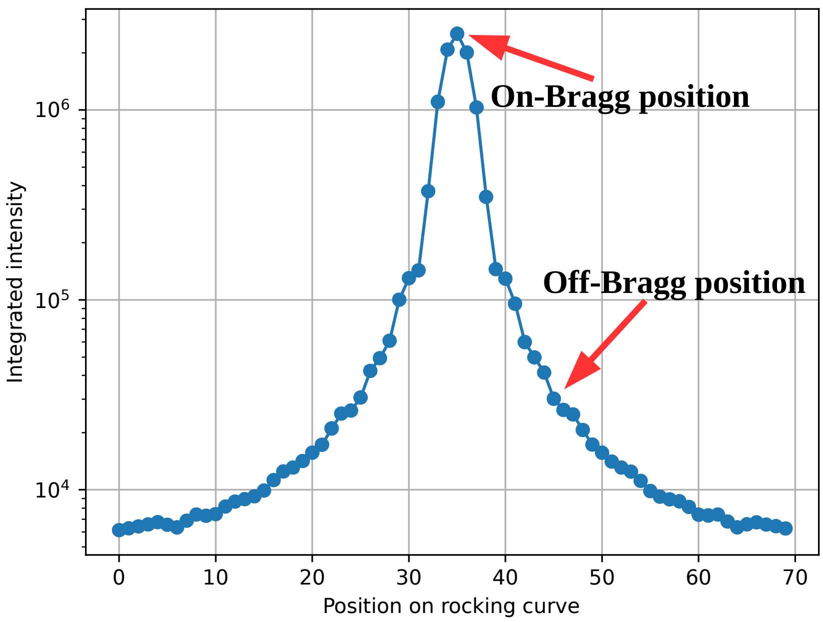

Figure 2: Integrated intensity of each slice along the rocking curve, showing the position of the on- and off-Bragg slices chosen for this demonstration.

one of which is the familiar "on-Bragg" central slice while the "off-Bragg" layer is 10 layers away form the central layer. These on- and off-Bragg layers were binned to mimic high-energy diffraction as described in Section 2 of the main text, followed by the addition of Poisson noise. Similar to the noise-free SRTF computation in the main text, the diffraction signal was upsampled along both diagonals of the detector.

This particular choice for the off-Bragg position represents the slice farthest from the Bragg peak (on either side) that we were able to recover the image from noisy simulated data. This corresponds to the boundary that includes two complete side-lobes in the integrated intensity of each slice along the rocking curve, as shown in Figure 2.

The pixel intensity of the ground truth was scaled to a maximum of $50,000$ counts (at the Bragg peak). This resulted in a signal-to-noise ratio (SNR) of $\sim$ 17dB for the off-Bragg layer and $\sim$ 50dB for the on-Bragg layer. The cutoff threshold for the on-Bragg layer was set to 5 counts in order to reduce the recovery artifacts. The results of the sparse recovery are shown in Figure 3.

As we can see from Figures 3c and 3f, reasonable approximations of the on- and off-Bragg layers can be recovered from diagonal detector offsets alone. Figures 3g and 3h show a similar trend of the SRTF towards the ideal valueo f 1, although there is far more variation for lower PBF and higher beam energies than the case without Poisson noise (refer to Figures 5c and 5d in the main text).

4. Different detector offsets

Here we demonstrate the ability of the sparse recovery algorithm to recover the signal with data from different combinations of detector offsets. We choose diffraction data binned at PBF = 6 and upsampled with detector offsets along both coarse pixel diagonals. For PBF = 6, there are 12 unique detector positions along these diagonals. Figures 4b, 4c, 4d, 4e show 4 of 10 different sets of randomized detector positions along the diagonals and Figures 4f, 4g, 4h, 4i denote the corresponding recovered images from the binned data thus obtained. As we can see from the histograms in Figure 4j, the SRTF was largely centered around 1 for upsampling recipes resulting from a variety of detector offset combinations. The images recovered from these different upsampling strategies are negligibly different from each other.

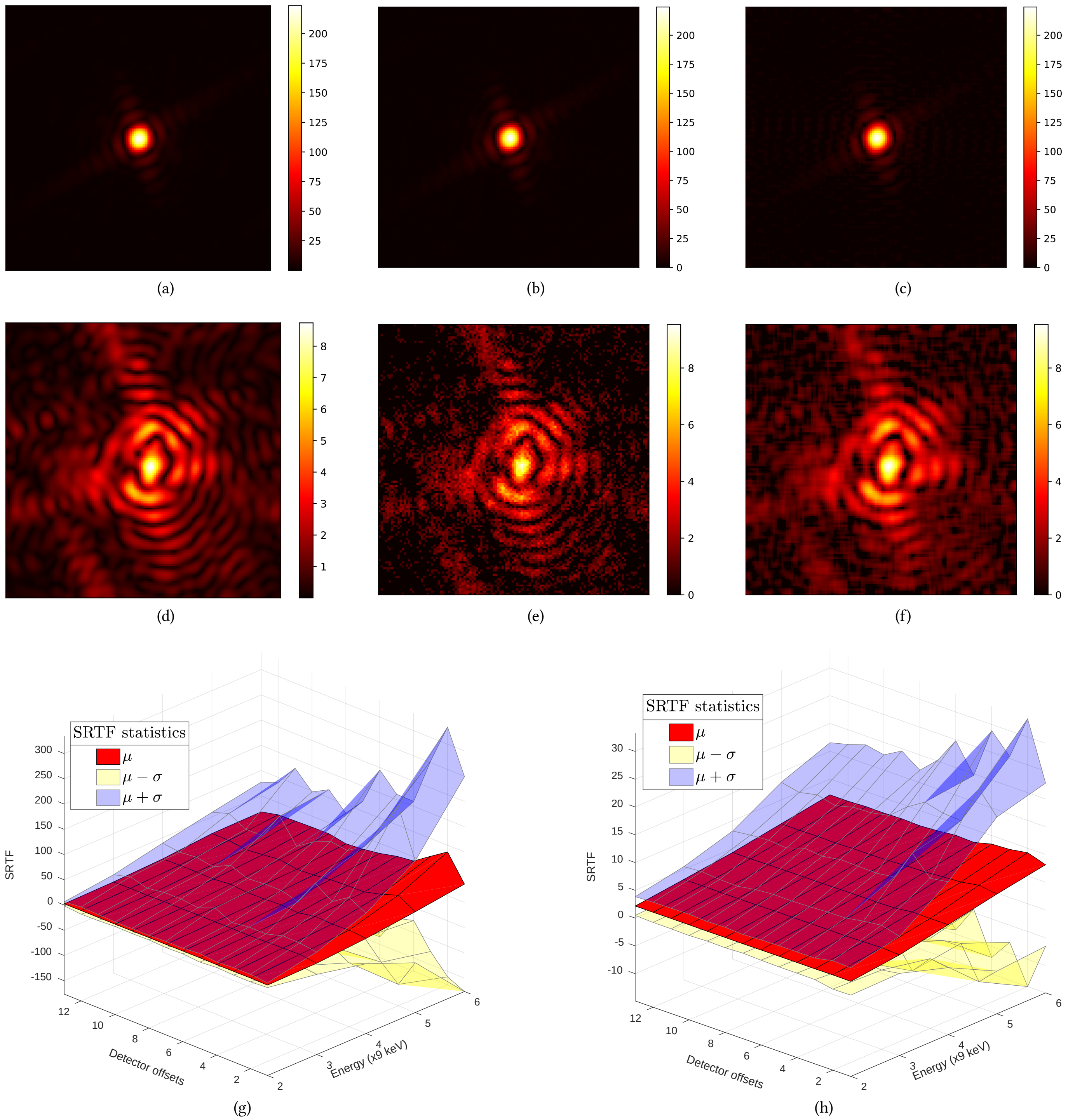

Figure 3: Scattering amplitudes $\left(\sqrt{I(i,j)}\right)$ for the **(a)** original on-Bragg image; **(b)** noisy on-Bragg image; **(c)** on-Bragg image recovered from binning at PBF = 6 and with 12 unique detector positions along the coarse pixel diagonals; **(d)** original off-Bragg image; **(e)** noisy off-Bragg image; **(f)** off-Bragg image recovered from binning at PBF = 6 and with 12 unique detector positions along the coarse pixel diagonals; **(g)** SRTF trend for on-Bragg image; **(h)** SRTF trend for off-Bragg image.

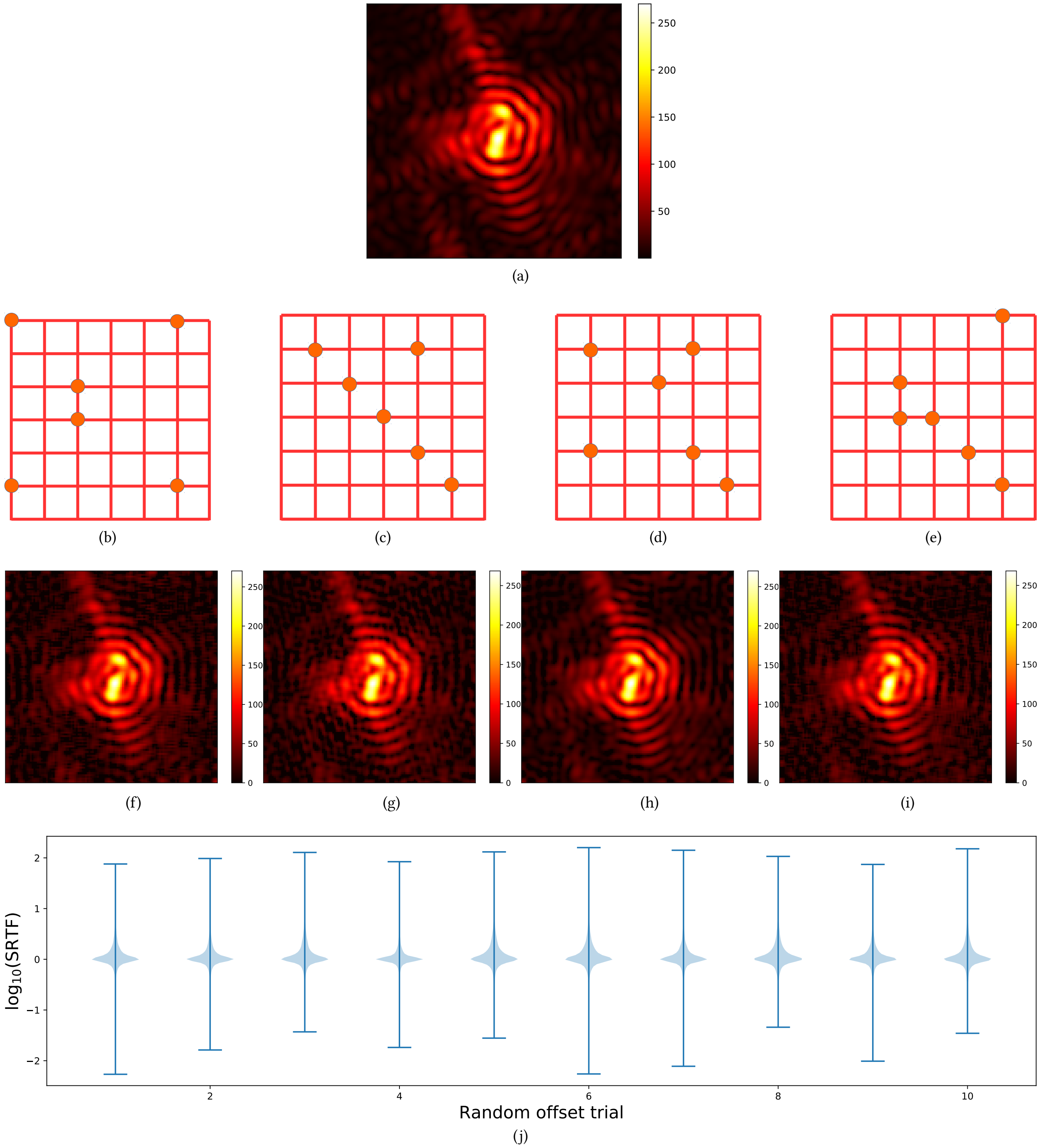

Figure 4: **(a)** Scattering amplitude $\sqrt{I(i,j)}$ of the original image; **(b),(c),(d),(e)** 4 differents sets of 6 detector offsets randomised along both coarse pixel diagonals. The circles denote the shifted position of the centre of the coarse pixel (*i.e* the central star in Figure 1b in Section 1 of this Supplementary Material; **(f),(g),(h),(i)** recovered scattering amplitudes corresponding to binned data obtained from each of the 6 sets of detector translations. **(j)** Histograms of the log of the SRTF for all 10 recovery trials. The error bars denote the extreme values.

5. References

[1] R. N. Bracewell. Strip integration in radio astronomy. *Australian Journal of Physics*, 9(2):198–217, Jun 1956.

[2] R. N. Bracewell. Numerical transforms. *Science*, 248(4956):697–704, 1990.
